# Molecular dynamics of DNA translocation by FtsK

**DOI:** 10.1093/nar/gkac668

**Published:** 2022-08-10

**Authors:** Joshua Pajak, Gaurav Arya

**Affiliations:** Dept. of Mechanical Engineering and Materials Science, Duke University, Durham, NC 27708, USA; Dept. of Biochemistry and Molecular Biotechnology, University of Massachusetts Chan Medical School, Worcester, MA 01605, USA; Dept. of Mechanical Engineering and Materials Science, Duke University, Durham, NC 27708, USA

## Abstract

The bacterial FtsK motor harvests energy from ATP to translocate double-stranded DNA during cell division. Here, we probe the molecular mechanisms underlying coordinated DNA translocation in FtsK by performing long timescale simulations of its hexameric assembly and individual subunits. From these simulations we predict signaling pathways that connect the ATPase active site to DNA-gripping residues, which allows the motor to coordinate its translocation activity with its ATPase activity. Additionally, we utilize well-tempered metadynamics simulations to compute free-energy landscapes that elucidate the extended-to-compact transition involved in force generation. We show that nucleotide binding promotes a compact conformation of a motor subunit, whereas the apo subunit is flexible. Together, our results support a mechanism whereby each ATP-bound subunit of the motor conforms to the helical pitch of DNA, and ATP hydrolysis/product release causes a subunit to lose grip of DNA. By ordinally engaging and disengaging with DNA, the FtsK motor unidirectionally translocates DNA.

## INTRODUCTION

Many vital biological tasks such as viral DNA packaging, protein degradation and chromosome segregation are completed by ring ATPase motors, which harness the energy from ATP binding, hydrolysis, and product release to translocate their biopolymer substrate through the central pore of the motor (1–6). These motors have evolved mechanisms to coordinate activities within and between individual subunits, so that the assembly functions as a cohesive unit rather than a collection of subunits operating independently (7–9). These mechanisms help all aspects of motor function. For instance, the motor must ensure that ATP is not prematurely hydrolyzed, that subunits are tightly gripping substrate before applying force, and that the resetting subunits are not tightly gripping substrate as they move in the direction opposite translocation. Although each aspect is likely controlled by distinct mechanisms, the motor nevertheless ensures that they occur in the proper sequence to yield unidirectional translocation of substrate. This implies that the motor has found ways to temporally connect individual mechanisms into one overall ‘well-oiled’ mechanism.

Understanding how ATPase motors transition between different structural states within this integrated mechanism has been a subject of intense investigation. Because motor translocation is a dynamic process, techniques like single-molecule force spectroscopy have been particularly useful to characterize intermediate states and the kinetics of transition (10–12). However, it is difficult to infer the underlying molecular mechanisms that produce force or promote coordination from single-molecule experiments alone. To this end, researchers have utilized cryogenic electron microscopy (cryo-EM) to obtain atomic structures of several ATPase assemblies actively translocating their substrates, which in most cases cannot be accessed by crystallographic methods. Recently obtained structures include the bacteriophage φ29 DNA packaging motor (13), katanin (14), 26S proteosome (15), Lon protease (16), Vps4 (17) and FtsK (18). Despite varying topologies and evolutionary divergences, each structure shows that the ATPase domains of actively translocating assemblies adopt helical arrangements as they engage with the biopolymer substrate. This strong conservation of quaternary structural arrangement, in addition to conservation of functional motifs (such as arginine fingers (8) and Walker motifs (19)), implies that there may be underlying mechanisms common to all translocating ring ATPase assemblies.

Among the aforementioned systems, bacterial FtsK is unique as it is the fastest DNA translocase currently known (20,21). Therefore, FtsK likely strikes a balance with respect to regulatory mechanisms, which introduce additional activation barriers, but simultaneously streamline the process by limiting off-pathway states. This makes the regulatory mechanisms in the FtsK motor informative to the broader class of ATPases. The FtsK translocase machinery is a homo-hexameric ring of ATPase subunits, where each subunit is further organized into domains. The α- and β-domains form the main translocase motor (called FtsK_αβ_), where the β-domains are the ATPase domains and DNA is translocated in the direction from the β-ring towards the α-ring.

Recently, Jean *et al.* (18) solved the cryo-EM structure of FtsK_αβ_ actively translocating double-stranded DNA, and showed that the motor is half occupied with the ATP analog and half occupied with ADP. ATP-analog-bound subunits were found to donate residues from ‘loop I’ and ‘loop II’ within the β-domain to grip opposite strands of DNA, whereas ADP-bound subunits do not. From this structure and additional biochemical evidence, they proposed a translocation model in which a hydrolysis event within a single subunit triggers conformational changes in every subunit. In this model, hydrolysis causes a subunit to disengage from DNA, permitting the other two upstream ATP-bound subunits to compact their α-/β-domains, thereby translocating DNA. At the same time, ADP release and ATP binding in a further upstream subunit causes that subunit to tightly grip DNA. These hydrolysis and nucleotide exchange events occur ordinally around the ring to yield continuous, step-wise translocation of DNA. The domain-level movements are strongly supported by the structural data and agree with previous characterization of this system (22–24). While the static structures offer valuable insight into the molecular interactions that motor subunits make with each other and with DNA, important questions about how these interactions give rise to the overall mechanism remain unknown. In particular, the nature of the driving force of subunit compaction during translocation, how subunits lose and gain registry with DNA based on nucleotide occupancy, and what mechanisms promote ordinal nucleotide exchange remain unknown.

Here, we used all-atom molecular dynamics (MD) simulations to investigate these open questions on molecular mechanisms governing DNA translocation by FtsK_αβ_. We advance the previously proposed model by predicting specific residue-level mechanisms that couple DNA gripping to ATP binding, subunit compaction to nucleotide occupancy, and additional *trans*-acting residues that may help promote ordinal ATPase activity. Finally, while a number of different mechanisms have been proposed for ASCE motors carrying out different tasks (25,26), we propose conservation of common functional elements across a broad class of molecular motors.

## MATERIALS AND METHODS

### Hexamer simulations

Initial structures were taken from the atomic model built into the cryo-EM reconstruction of hexameric FtsK_αβ_ motor assembly captured in the middle of translocation, containing three ADP- and three ATP-γS-bound subunits (PDB: 6T8B) (18). ATP-γS was manually changed to ATP. Missing loops (e.g. residues ∼570–585) were rebuilt using MODELLER (42), using the high-resolution crystal structure of FtsK_αβ_ (PDB: 2IUU) as a constraint. Mg^2+^ was added to ATP/ADP binding sites where it was not built.

Simulations were performed with the GPU-accelerated *pmemd* module of the AMBER20 simulation package (43). The protein interactions were described using the AMBER ff19SB force field (44) with the OPC water model (45). DNA was described using the OL15 modifications (46) to the ff99bsc0 parameters (47,48). ATP and ADP parameters were taken from the AMBER parameter database (49). The hexamer assembly was centered in a truncated octahedron box of OPC water with 14 Å padding. Counter ions were added to make the system charge-neutral, and then additional salt was added to reach 150 mM concentration. The system had a total of ∼288,000 particles; the AMBER topology file and initial coordinates are included in the supplementary data. To enhance computational performance, hydrogen mass repartitioning (HMR) was applied with ParmEd (50), increasing the solute hydrogens mass to 3.024 Da, allowing for a 4-fs integration timestep. The system was subjected to 300 steps of steepest descent and conjugate gradient energy minimization. The SHAKE algorithm (51) was used to constrain bonds connecting hydrogen atoms to heavy atoms. Duplicate simulations were seeded by drawing different randomized initial velocities. The systems were heated from 100 to 310 K over the course of 100 ps in the canonical (NVT) ensemble, while restraining the protein backbone, DNA phosphates, and ATP/ADP phosphates with a 10 kcal/mol·Å^2^ harmonic potential. During heating, a 2-fs integration timestep was used to propagate the equations of motion. Then the production runs were performed with no positional restraints in the isobaric-isothermal (NPT) ensemble at 310 K and 1 bar using the Monte Carlo barostat and Langevin thermostat with a 2.0 ps^–1^ collision frequency and 1.0 ps relaxation time (52–54). During production, a 4-fs integration timestep was used to propagate the equations of motion. The Particle-Mesh Ewald (PME) scheme was used to correct long-range interactions with a 9 Å explicit cutoff. Production runs were performed for 1 μs. AMBER .in files for minimization, heating, and production are included in the supplementary data.

### Monomer simulations

Monomer simulations were started by isolating specific subunits from the hexameric complex: ATP-bound simulations started with an isolated ATP-bound subunit, and ADP-bound simulations started with an isolated ADP-bound subunit. The apo state simulations started from an ATP-bound subunit where Mg^2+^-ATP was manually removed from the binding pocket, hence, any differences predicted between ATP-bound and apo simulations are a direct result of nucleotide occupancy and not initial structures. Topology files and initial coordinates are included in the supplementary data.

As above, we chose to describe monomer systems with the recent AMBER ff19SB force field (44) with the OPC water model (45). The systems were centered in truncated octahedral periodic boxes with OPC water molecules with 14 Å padding. Hydrogen mass repartitioning was not applied to the monomer simulations. In all other respects, the systems were energy-minimized and duplicate simulations were equilibrated as described above with a 2-fs integration timestep. Production simulations were run for 1 μs each, and trajectories were saved every 25 ps.

### Metadynamics simulations

Well-tempered metadynamics simulations were performed using PLUMED (55) as interfaced with the AMBER18/AMBER20 simulation packages. The collective variables were chosen to be the extension and twist angles described earlier. Specifically, *extension* is defined to be the angle that connects the centers of mass of residues 376–391, 424–428 and 657–664. *Twist* is defined as the dihedral angle that connects the centers of mass of residues 376–391, 362–365, 424–428 and 657–664. Starting structures were taken from equilibrium MD simulations described after 500 ns of sampling. Gaussian hills were deposited every 500 integration timesteps with initial heights of 1.5 kJ/mol and a bias factor of 15. For the monomer-DNA complex system, we used multiple-walker metadynamics with twelve walkers exploring the collective variable space. Metadynamics simulations were considered converged when all the collective variable space had been sampled and free energy landscapes achieved asymptotic behavior with respect to number of hills included in the calculations. PLUMED input files are provided in the supplementary data.

### Post-simulation analysis

Clustering was performed with the cpptraj package (56) (*k*-means, *k* = 10) for each simulation; a representative frame from the most dominant cluster is provided as a PDB file in the supplementary data; these PDB files have been manually renumbered for ease of viewing. Principal components of motion were calculated using the ProDy (57) module in VMD (58); .nmd files that visualize these projections are included in the supplementary data. We additionally performed PCA on the first half and second half of our monomer simulations, which show good agreement with each other (Supplementary Figure S2), suggesting that these results are converged. Mutual information was calculated using the CARDS method (59) implemented in the Enspara package (60). Frames from ATP-, ADP-bound and apo simulations were aggregated into the CARDS analysis; this allows the mutual information to capture conformational changes associated with ATP hydrolysis and ADP release. We calculated CARDS using half data sets (e.g. one ATP-, one ADP-bound, and one apo simulation) and compared those results to the overall calculated MI, showing good agreement (Supplementary Figure S5). Target site analysis was used to isolate the mutual information to locations of interest, as discussed in the results. VMD (58) and UCSF ChimeraX (61) were used for visualization. The Julia language (62) and the Seaborn (63) Python library were used to create plots.

## RESULTS

### ATP binding engages DNA gripping by loop II of the β-domain

To investigate conformational changes induced by bound nucleotide that contribute to DNA translocation, we performed microsecond-long MD simulations of subunit monomers in the apo, ATP- and ADP-bound states. By understanding how an individual subunit responds to nucleotide occupancy, we can better understand how an assembly of subunits may respond to binding, hydrolysis, and product release at a single subunit.

**Figure 1.**
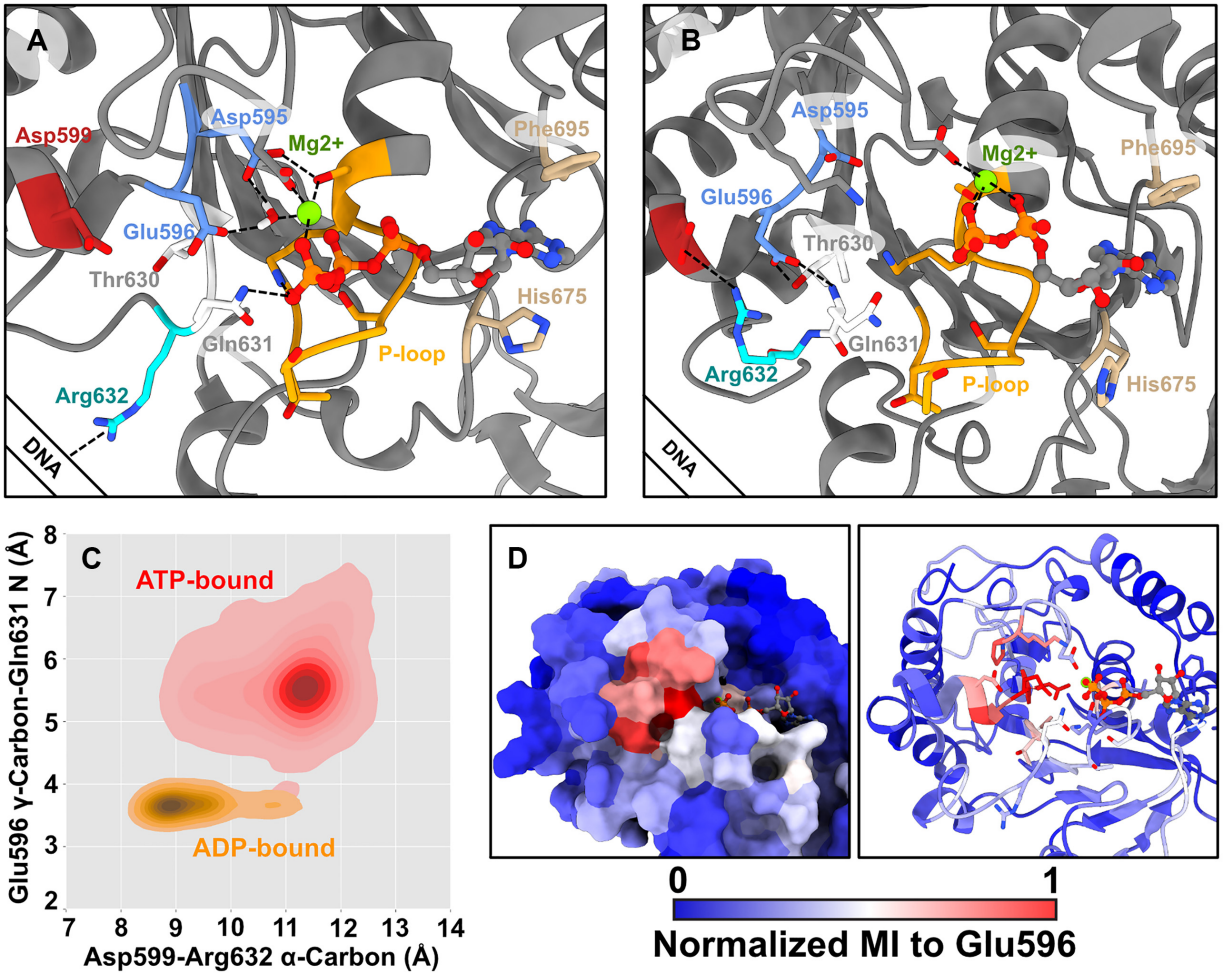
ATP binding causes loop II to grip DNA. (**A**) A representative conformation from the largest cluster of our ATP-bound simulations. The Walker A motif (P-loop) is orange, Walker B is cornflower blue, phosphosensor motif is white, DNA-gripping loop II Arg632 is cyan, Asp599 is dark red, and residues that π- stack with adenosine are tan. While DNA is not included in the simulation, we depict the general location of DNA relative to the subunit. A bound water molecule is held by the Walker B motif. (Not shown) A sodium ion acts in place of trans-acting residues, which has been previously observed in simulation of ATPase monomers isolated from larger assemblies (27). (**B**) A representative conformation from the largest cluster of our ADP-bound simulations. The catalytic Glu596 now hydrogen bonds with the phosphosensor motif Thr630 and Gln631, and loop II Arg632 rotates inward to interact with Asp599, such that it is less likely to interact with DNA. (**C**) Distribution of distances from duplicate microsecond-long simulations. In ATP-bound simulations, the catalytic Glu596 does not interact with the backbone of Gln631, and the backbone of Asp599 is far from Arg632. As Glu596 hydro-gen bonds to the backbone, it helps pull Asp599 close to Arg632, promoting their interaction. (**D**) Normalized mutual information to the catalytic glutamate high communication with other residues expected to coordinate γ-phosphate binding, and that the MI decays across the Walker A motif. Thus, this coordination cluster is uniquely poised to respond to the hydrolysis of ATP and departure of the inorganic phosphate to control the subunit's grip on DNA.

The simulations revealed two predominant conformations of the ATPase active site depending on the state of the bound nucleotide. In the ATP-bound simulations, the catalytic glutamate (Glu596) points its carboxylate group towards the bound nucleotide (Figure 1A) with a localized water molecule. Previous studies on AAA+ and viral packaging ATPases have identified this conformation of the catalytic glutamate residue to be poised for catalytic activity (9,28,29). However, in the ADP-bound (as well as apo) simulations, Glu596’s carboxylate group instead hydrogen bonds with the phosphosensor motif (Thr630 and Gln631) (Figure 1B, Supplementary Figure S1). A result of these interactions is a decreased distance between the backbone of the catalytic glutamate and phosphosensor motifs, bringing DNA-gripping loop II Arg632 closer to Asp599 (Figure 1C). In the ATP-bound simulations, Arg632 points away from the surface of the protein, such that it can potentially engage with substrate DNA (Figure 1A); in the apo and ADP-bound simulations Arg632 is flush against the surface of the protein, interacting with Asp599, and would therefore not be expected to engage with DNA (Figure 1B).

The dependence of the catalytic glutamate on the presence/absence of the γ-phosphate of ATP to control DNA-gripping is reminiscent of the ‘glutamate switch’ found in AAA+ enzymes (9,28). In this case, the phosphosensor motif is acting in place of the ‘canonical’ glutamate switch to hydrogen-bond with the catalytic glutamate. Importantly, the phosphosensor motif is immediately upstream of a key DNA-gripping residue, Arg632, suggesting a simple line of communication between the active site and DNA-gripping residues.

We previously predicted from mutual information (MI) extracted from MD simulations that viral DNA packaging enzymes use a glutamate switch to couple ATPase and DNA packaging activities, and this prediction was corroborated by functional mutagenesis (29). Inspired by these results, we extracted MI from our monomer simulations using the Correlation of All Rotameric and Dynamical States (CARDS) method. In general, MI is a method to understand how dependent two variables are on each other by leveraging their joint and marginal distributions. In this case, CARDS considers each residue as a variable whose value is the state determined by its backbone Ramachandran angles and rotamer. A residue's state and transitions between states is correlated with all other residues in the protein; if two residues have high MI, we infer communication between these residues. Our simulations revealed that the catalytic glutamate has high MI with several residues that are expected to coordinate the γ-phosphate of ATP (Figure 1D). Additionally, this MI extends to Asp599, Thr630 and Gln631, which can control the position of Arg632. We find a sharp decrease in MI across the binding site, indicating that the catalytic glutamate is not strongly affected by residues that bind other parts of ATP/ADP, reinforcing the idea that these residues are uniquely situated to respond to ATP hydrolysis. Thus, from both our distance/hydrogen-bonding analysis and our MI analysis, our MD simulations predict that nucleotide-occupancy is sensed by the catalytic residues and relayed to loop II DNA-gripping residues, namely Arg632, to control grip of DNA. Our simulations predict a feasible mechanism for how FtsK regulates its grip on DNA pre- and post-hydrolysis that is consistent with other translocating ATPases.

### Rotations of the α- and β-domains are the primary modes of motion

**Figure 2.**
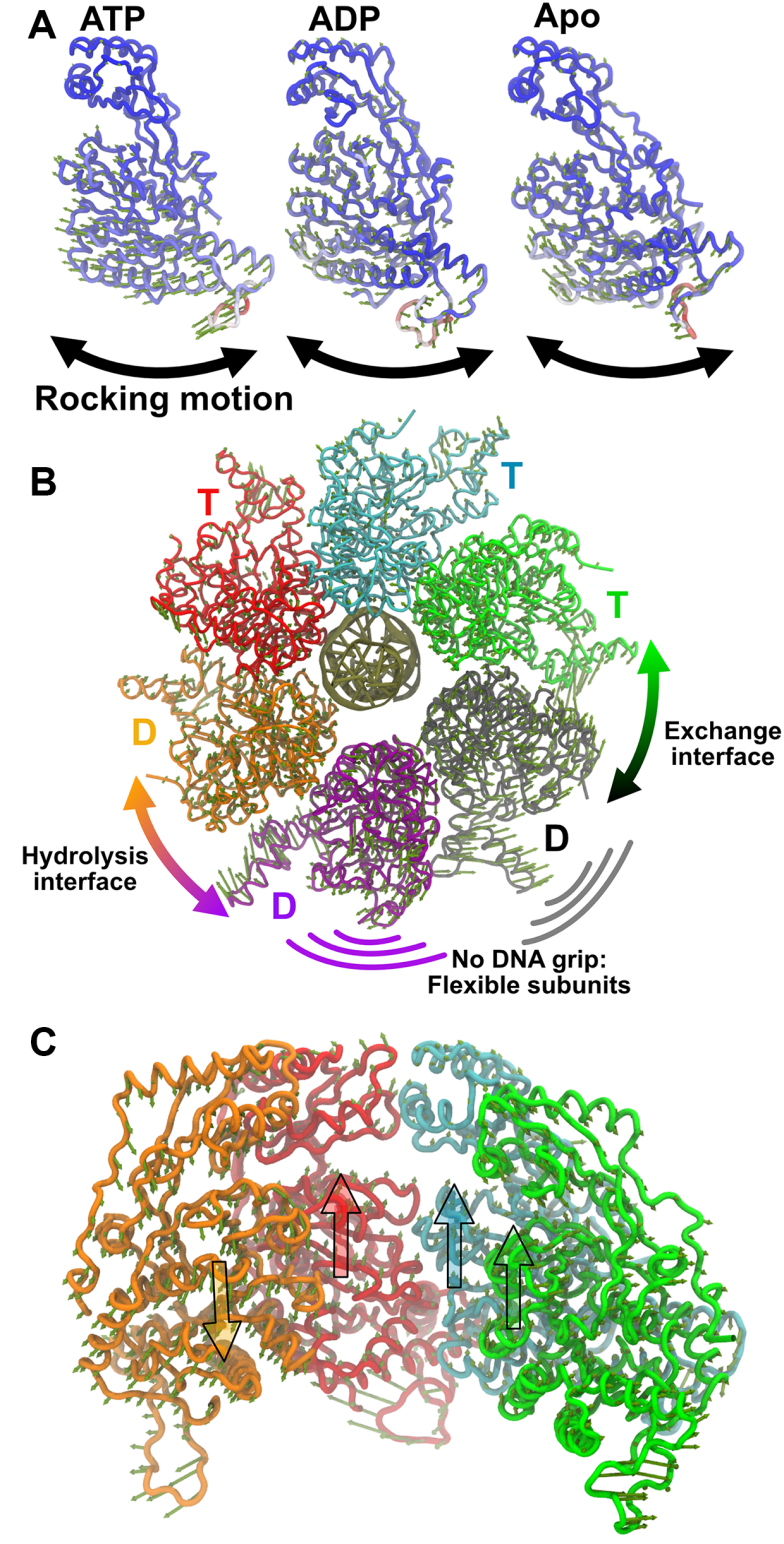
Principal components of motion. (**A**) The first principal component of motion is depicted for the ATP-, ADP-bound, and apo monomer simulations after aligning about the α-domain. Residues are colored according to flexibility, with more flexible residues as red and more rigid residues as blue. The predominant motion in every case is a rocking of the β-domain relative to the α-domain, indicated by the bold arrows. (**B**) The first principal component of motion from the hexamer simulations shows that the two ADP-bound subunits that do not grip DNA (purple and gray) are highly flexible. This flexibility allows the motor to easily sample states around the interface where hydrolysis presumably occurs, and the interface where nucleotide exchange occurs (see also Movie S7). (**C**) The first principal component of subunits in the β-domain helix shows that the ATP-bound subunits that tightly grip DNA move their domains sympathetically, while the ADP-bound subunit moves oppositely (see also Movie S8). Large translucent arrows are depicted to help guide the eye. This suggests that tightly gripping DNA coordinates the motions of ATP-bound subunits, allowing them all to contribute towards DNA translocation past the hydrolyzing subunit.

We calculated the principal components of motion from our monomer simulations and found that the dominant motion is a rocking of the β-domain relative to the α-domain (Figure 2A, Supplementary Figure S2, Movies S1–6). This is perhaps expected, as the two domains are connected by a flexible linker and this motion was proposed to be responsible for translocating DNA based on structural evidence (18). However, while monomer dynamics can provide insights into the predominant motions of a subunit, these motions can be altered by interactions with neighboring subunits and DNA. Thus, in addition to our monomer simulations we also performed long timescale MD simulations of the hexameric assembly with DNA in the central pore. PCA of the entire hexameric complex predicted that subunit motions are coordinated through their association with DNA. The three ATP-bound subunits that grip DNA rock their domains sympathetically, whereas the ADP-bound subunit at the top of the β-ring helix moves oppositely as it loses tight grip of DNA (Figure 2C, Movie S7). This suggests that ATP-bound subunits translocate DNA past the hydrolyzing subunit as it begins to lose grip of DNA and reset to the other side of the β-ring helix. Thus, the ATP-bound subunits maintain helical registry with DNA, which allows these tightly gripping subunits to cooperatively translocate DNA.

Meanwhile, the ADP-bound subunits that do not track DNA (purple and gray) are less-restricted in their motion. Across both simulations, the primary mode of motion captures rotation of these two subunits that affects how their ATPase active site interacts with neighboring subunit's *trans-*acting residues and *vice-versa* (Figure 2B, Movie S8). This is of note, as the active sites that are primarily affected by these rotations are the sites where hydrolysis and nucleotide exchange putatively occur (this is discussed further below). Thus, the FtsK motor may leverage subunit flexibility imparted by not gripping DNA to promote important catalytic functions at specific subunit interfaces.

**Figure 3.**
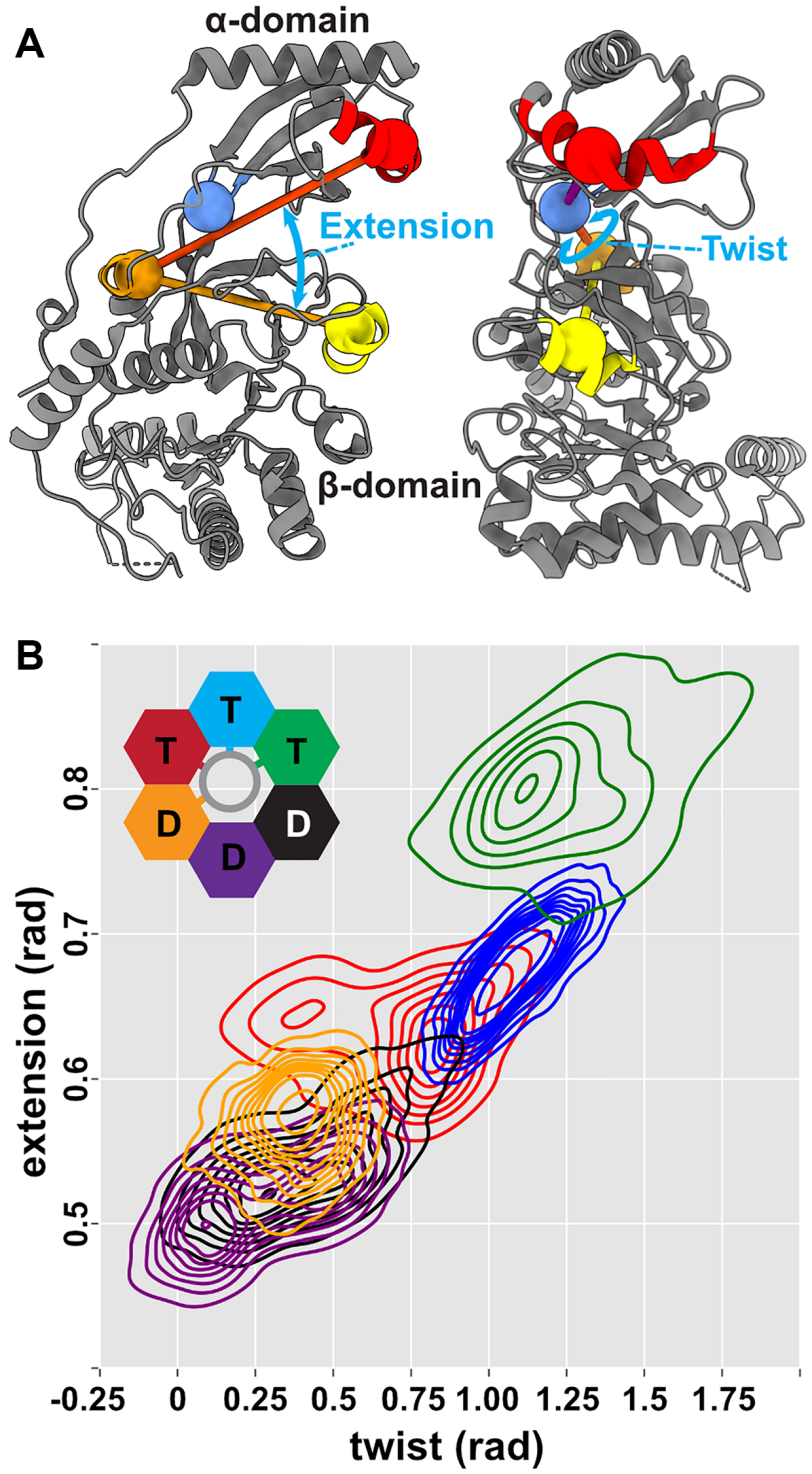
Collective variables that characterize motion. (**A**) The extension angle describes the separation of DNA-gripping helices in the α- and β-domains. The twist angle describes the shearing between these helices. (**B**) Subunit conformations sampled in one hexamer simulation projected on the twist and extension angles. DNA tracking subunits (green, cyan, red, and orange) form a continuous distribution. Subunits that do not grip DNA (purple and gray) do not necessarily follow this trend, as they are not constrained by interaction with DNA. See Supplementary Figure S3 for the distribution from the second simulation.

Next, we investigated the principal components of motion of individual subunits within the hexamer from the DNA-bound simulations. Our analysis predicted that, like their monomeric counterparts, dynamics of a monomer within the hexamer are also dominated by rocking of the α- and β-domains (Movie S9). The first principal component of motion also encompasses a shear between the α- and β-domains. Based on this observation, we defined two collective variable angles: *extension*, the angle that describes the separation between DNA-gripping motifs in the α- and β-domains, and *twist*, the angle that describes the shearing between the domains (Figure 3A). We then projected our hexamer simulations onto these two collective variables and found that there is a continuous distribution of states sampled by the hexamer (Figure 3B, Supplementary Figure S3). The subunits with the largest extension and twist are those bound to ATP, which track the helical DNA substrate. The subunits with less extension and twist are ADP bound, which are largely not constrained by interaction with substrate DNA.

### Nucleotide binding promotes compaction

**Figure 4.**
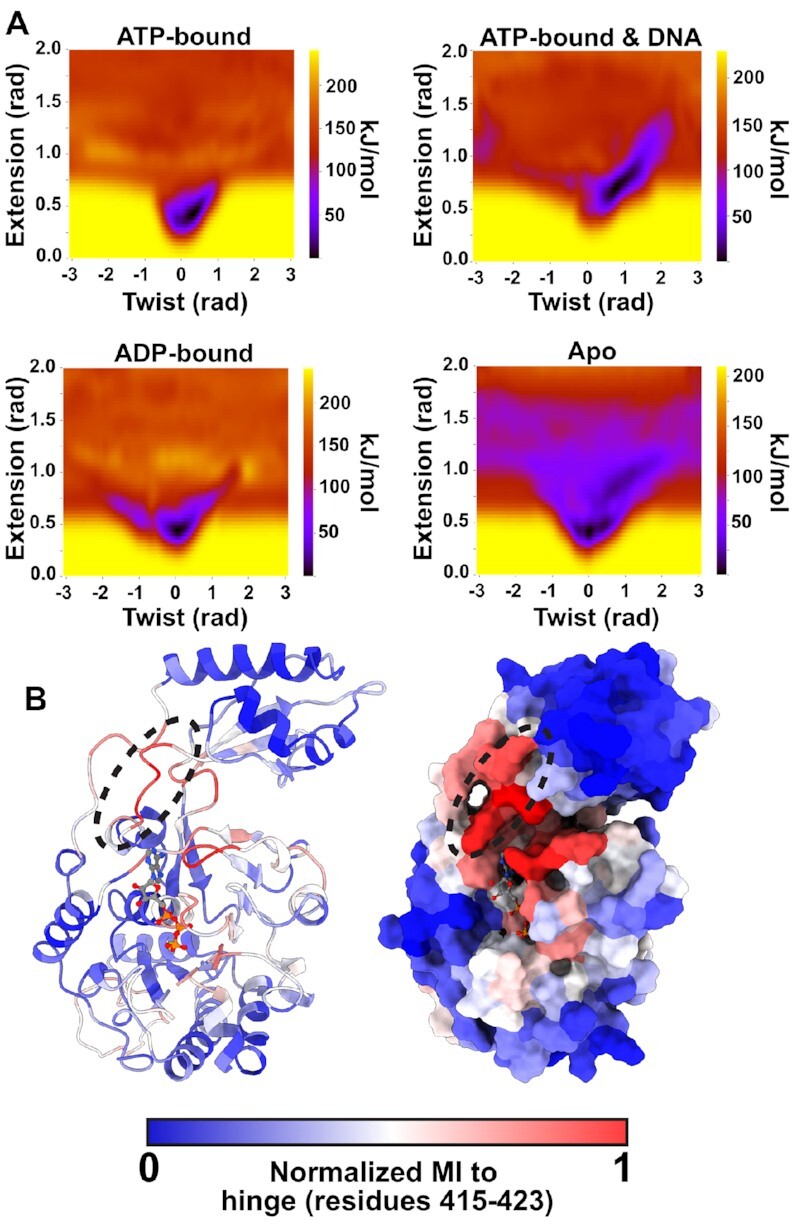
Free-energy and mutual information calculations predict that nucleotide compacts subunits. (**A**) Free-energy landscapes in the twist-extension collective variable space calculated by well-tempered metadynamics. In the nucleotide-bound states, the compact state (low extension) is the global free-energy minimum. In the apo state, while the compact state is the global minimum, the collective variable space is much more accessible, with metastabilities emerging in twisted and extended conformations. DNA stabilizes twisted and extended conformations in the ATP-bound state as the subunit tracks the helical phosphate backbone. The calculated free-energy minimum corresponds well with the states sampled in by DNA-gripping subunits in the hexamer simulation (Figure 3B). (**B**) Mutual information calculated by CARDS, targeting the hinge connecting the α-/β-domains. Hinge (dashed circle) and interface residues have high mutual information to residues that bind the adenosine and α-/β-phosphates of ATP. Notably, this mutual information decays substantially prior to reaching the Walker B motif, which primarily responds to the γ-phosphate of ATP (Figure 1), suggesting that compaction is a nucleotide-binding dependent and hydrolysis- independent process.

To further probe the connection between nucleotide occupancy and preferred relative orientations of the α- and β-domains, we calculated free-energy landscapes of a subunit in the extension and twist collective variable space using well-tempered metadynamics simulations. Our calculations predict that both ATP- and ADP-bound subunits prefer a compact conformation (Figure 4A). On the other hand, the apo state subunit samples relatively broad collective variable space with little energetic penalty (Figure 4A). In other words, the nucleotide-bound states are predicted to be rigid and compact, whereas the apo state is predicted to be flexible.

Next, we wondered how interaction of a subunit with DNA could affect this free-energy landscape. Thus, we likewise calculated the free-energy landscape of an ATP-bound subunit complexed with DNA. We found that interaction with DNA stabilizes the more twisted and extended conformations (Figure 4A). The shape and span of this free-energy minima valley agrees well with the states sampled by ATP-bound monomers in our hexamer simulation (Figure 3B). Thus, the protein-DNA interactions could stabilize ATP-bound subunits in twisted and extended conformations.

Lastly, to determine how nucleotide occupancy might control the conformation of the subunit, we extracted mutual information from our equilibrium MD simulations. We performed a target site analysis relative to the flexible linker (or hinge) connecting the α-domain to the β-domain, reasoning that controlling the linker between the domains could provide a means to control the relative orientations of the two domains. We find that this hinge has high MI to residues involved in binding the adenosine ring (e.g. His675 and Phe695) and the α-/β-phosphates (e.g. the Walker A motif/P-loop) (Figure 4B). Notably, this MI decays substantially as it reaches the Walker B motif, which we demonstrated above primarily responds to the presence or absence of the γ-phosphate of ATP (Figure 1D). Thus, the shared information is coordinated by ATP and ADP alike, explaining why both nucleotides drive compaction.

### Geometry of the β-ring promotes ordinal activity

Based on the solved cryo-EM reconstruction of actively translocating FtsK_αβ_, the most commonly observed state of the motor is contiguous stretches of three ATP-bound and three ADP-bound subunits. Because the class-averaged structure is dominated by this one configuration despite potentially stalling at any translocation step, it was inferred that each translocation step results in a rotationally symmetric configuration (18). Additionally, during translocation, subunits must release ADP to bind ATP. This implies that the motor has evolved mechanisms that selectively promote ordinal ADP release (and subsequent exchange with ATP) in one subunit and not the other two ADP-bound subunits, otherwise different ATP/ADP nucleotide permutations would have been observed.

**Figure 5.**
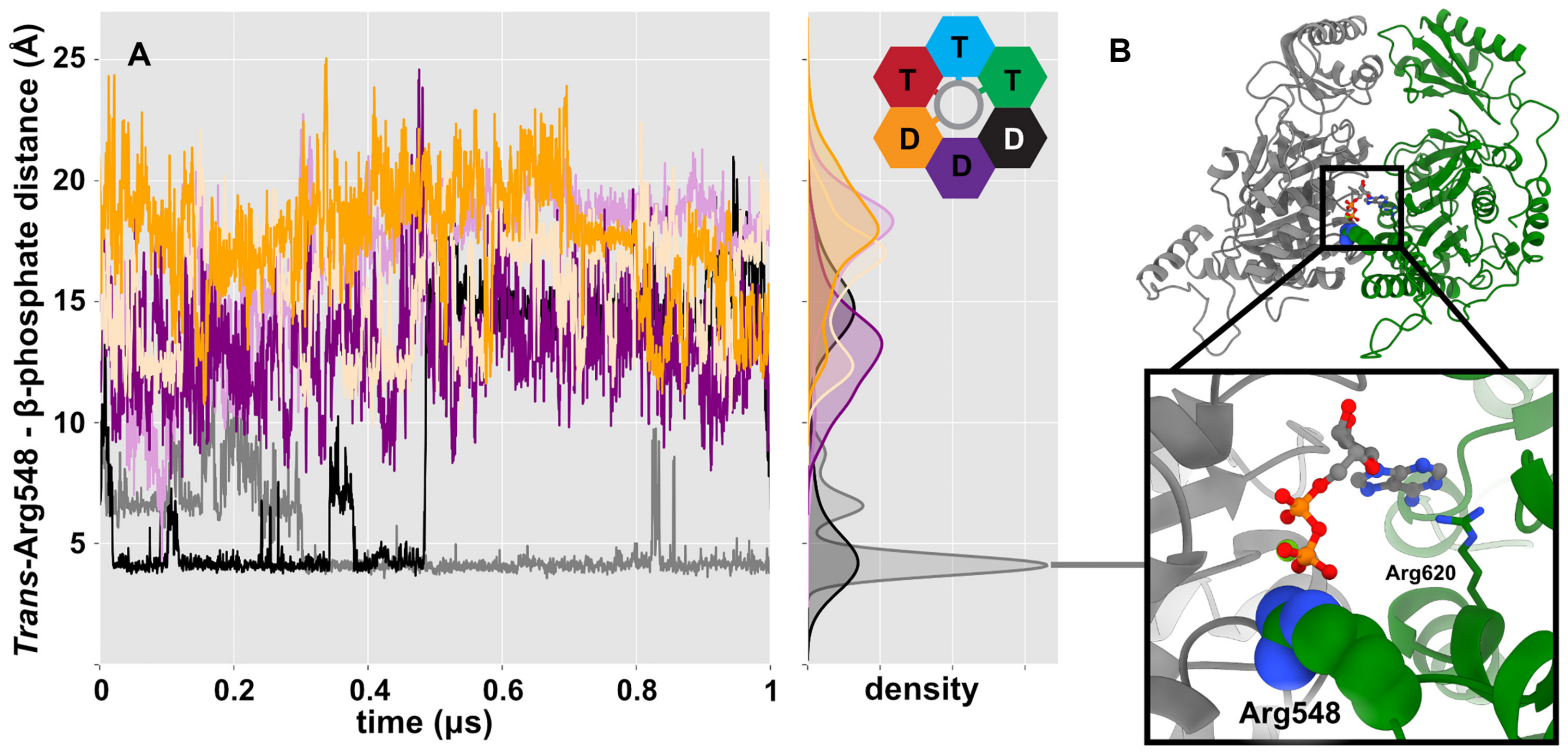
Geometry of the ring promotes ordinal ADP release. (**A**) Distances between the guanidine carbon of Arg548 and the β-phosphate of ADP bound to the neighboring subunit. Colors correspond to the cis-acting binding site; pastel and dark designate duplicate simulations. Arg548 only interacts closely with the β- phosphate of the subunit next in line to exchange ADP for ATP, suggesting that it helps promote nucleotide exchange. (**B**) A depiction of this interaction, corresponding to a representative frame from the largest cluster of a hexamer simulation and the density peak indicated by the gray line. The canonical arginine finger Arg620 is also shown.

Our MD simulations of the hexameric assembly with substrate DNA sheds light on one possible mechanism. At the proposed nucleotide exchanging interface (and not in the other ADP-bound interfaces) additional *trans*-acting residues interact with the bound ADP (Figure 5A, green subunit donating *trans*-acting residues into the black subunit). Specifically, we find that Arg548 interacts with the phosphates of ADP (Figure 5B). These interactions might destabilize the interactions between ADP and *cis*-acting residues, thus potentially promoting ADP release and subsequent ATP binding.

The interactions between Arg548 and the neighboring subunit's ADP seem to be enforced by the geometry of the ring. The residues are donated by the most extended ATP-bound subunit. Because subunit extension is not a simple linear translation, but rather is accomplished by rocking the β-domain relative to the α-domain, there is a secondary effect of extension pushing *trans*-acting residues upwards and towards the neighboring subunit (Figure 2 and Movie S3). Thus, a subunit binding ATP and engaging with DNA at the bottom of the β-ring helix positions Arg548 to act as an exchange residue, promoting ADP release and ordinal nucleotide exchange.

A similar mechanism could potentially ensure ordinal ATP hydrolysis events. In the cryo-EM reconstruction, the canonical arginine finger (Arg620) is near to the γ-phosphate of ATP in every ATP-analog-bound subunit. Hence, it is not readily apparent why hydrolysis would only occur in one specific subunit and not the other two ATP-bound subunits. In our hexamer simulations, Arg548 can also transiently interact with the γ-phosphate of ATP in addition to the canonical arginine finger interaction (Supplementary Figure S4). Although these interactions are much less stable than the ADP-bound interaction shown in Figure 5 and are at an unexpected interface, it nevertheless demonstrates the possibility for this residue to contribute towards hydrolysis. Thus, we also propose that this second arginine residue may play a catalytic role in addition to the canonical arginine finger. This analysis together with our PCA results (Figure 2) suggests that the FtsK motor may capitalize on both the unique geometry of specific interfaces and the inherent flexibility of subunits that do not grip DNA to promote catalytic and exchange activities.

## DISCUSSION

### Proposed mechanism of DNA translocation by FtsK

**Figure 6.**
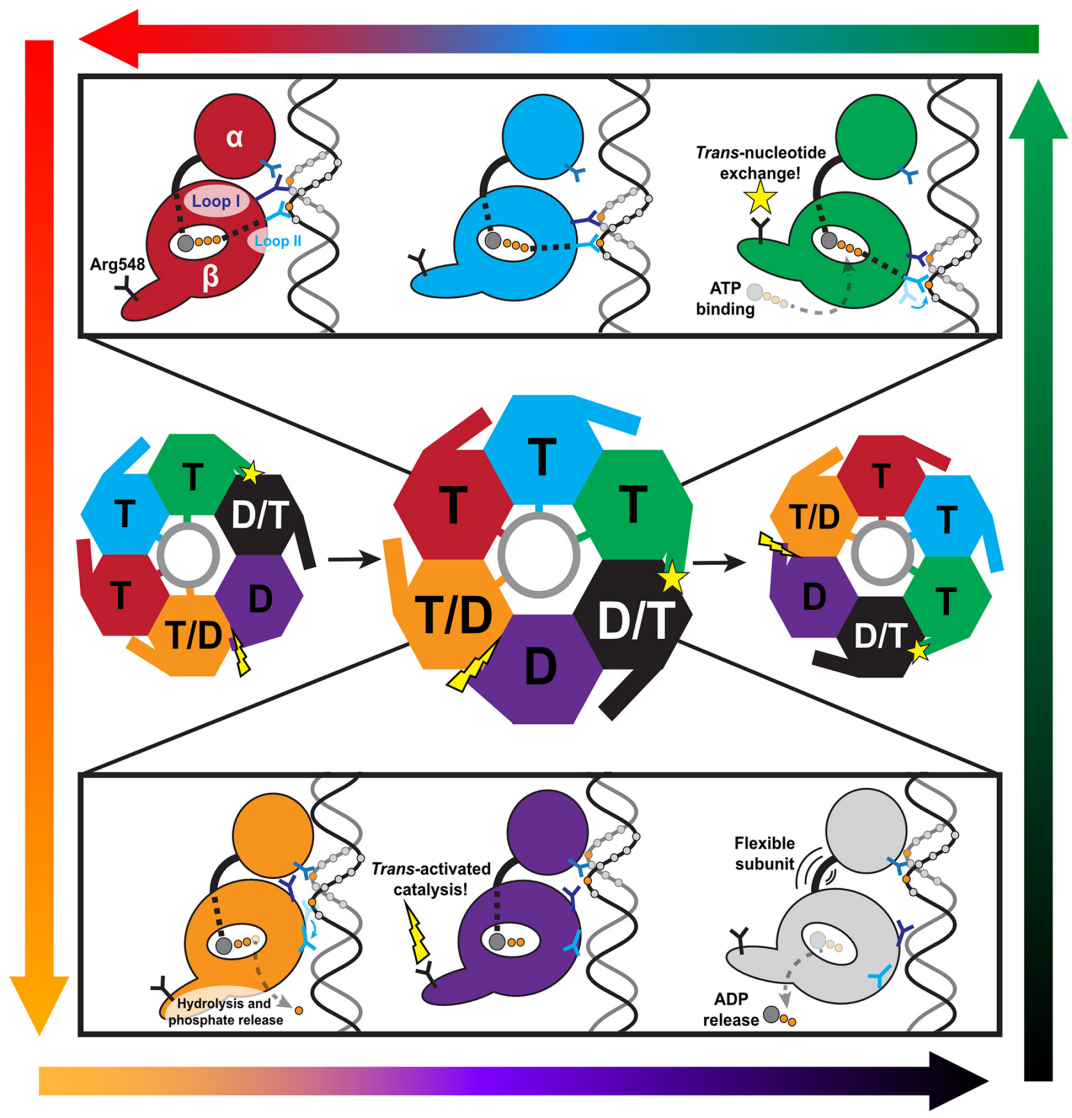
Overall mechanochemical cycle of DNA translocation by FtsK. The overall mechanochemical model is based off the one proposed in Jean et al. (18). Here, dynamic information taken from MD simulations is used to flesh out the individual steps in the cycle. In the center, the conformational changes of the hexameric complex are depicted. Subunits are labeled ‘T’ if they are ATP-bound, ‘D’ if they are ADP-bound. We label a subunit ‘T/D’ if we expect that subunit be catalytic (hydrolyzing ATP to ADP), and ‘D/T’ if we expect that subunit to exchange ADP for ATP. Subunits are colored according to their state in relation to their neighbors and the overall helical pitch of the motor. As the motor hydrolyzes ATP and exchanges ADP, these relative positions permute around the complex (the complex itself does not rotate). *Trans*-catalyzed ATP hydrolysis and nucleotide exchange are shown by a lightning bolt and star, respectively. Contacts with DNA (gray circle) are shown as sticks. In the top panel, the ATP-bound subunits are depicted. Signals sent from the bound nucleotide to Loop II and the flexible hinge are shown as dashed lines. For simplicity, the multiple DNA contacts in Loop I, Loop II, and the α-domain are shown as singular prongs. In the bottom panel, ADP-bound subunits are shown, and the ‘D/T’ subunit is shown as passing through the transient apo state. In the top and bottom panels, some DNA phosphates are shown as circles colored orange if they were in registry with the subunit and gray if they were not. These phosphates guide the eye to DNA translocation as a single subunit begins at the bottom of the ATP helix (green); works its way towards the top while remaining in registry with and translocating DNA (cyan, red); hydrolyzes ATP and loses registry in the β- domain (orange); and resets to the bottom of the β-domain helix while the subunit is not engaged with DNA (purple and gray), finally realigning with the phosphate backbone and beginning the cycle over again (green). This order of events is guided by the gradient arrows encircling the figure.

The work presented herein expands upon the model proposed in Jean et al. (18). In the proposed model, simultaneous conformational changes occur around the ring coupled to ATP hydrolysis at the ‘top’ of the β-ring and nucleotide exchange at the ‘bottom’ of the β-ring (Figure 6, orange and black subunits). These conformational changes cycle the overall assembly configuration one subunit around the ring. However, several fundamental questions remain about the molecular nature of these events. Our results allow us to address these questions by proposing specific molecular mechanisms that coordinate activities across subunits and generate force necessary to translocate DNA.

We begin our description of the model at the configuration solved in Jean *et al.* (18). In this configuration, three ATP-bound subunits’ β-domains track DNA substrate. Our simulations show that while bound nucleotide drives subunits into a compact conformation, the extended subunits at the bottom of the β-ring twist and are stabilized by interactions with the double-stranded DNA substrate (Figure 4). At the top of the β-ring helix sits the hydrolyzing subunit, which was experimentally solved to be in the ADP-bound state (18). We speculate that this interface is immediately post-hydrolysis because the neighboring subunit, which donates catalytic residues *in trans*, does not grip DNA and is more flexible (Figure 2). Thus, it can more readily sample states that will promote ATP hydrolysis in this subunit and differentiates this interface from the other ATP-bound interfaces. ATP hydrolysis signals DNA-gripping loop II to disengage with DNA (Figures 1, and 6). Thus, upon reaching the top of the β-ring helix, hydrolysis causes the subunit to lose registry with substrate DNA in its β-domain. In our simulations and the cryo-EM reconstruction alike, subunits also grip DNA with their α-domains. Therefore, we speculate that these additional grips stop DNA from backsliding as subunits lose grip in their β-domains and begin to reset.

At the other end (bottom) of the β-ring helix, ADP release is promoted *in trans* by an ATP-bound subunit (Figures 5, and 6). Again, because this subunit (gray) is not gripping DNA and is flexible (Figure 2), it can more easily sample conformations that will promote nucleotide exchange. The extended ATP-bound subunit (green) rocks its β-domain (Figure 2) as it extends to the bottom of the β-domain helix, placing Arg548 close to the departing ADP. Thus, the flexibility and unique geometry of this interface help to selectively promote nucleotide exchange. During the transient apo state, this subunit becomes highly flexible (Figure 4A), allowing it to extend to the bottom of the β-ring helix, potentially promoted by nascent interactions with DNA phosphates. Subsequent ATP binding causes this subunit to engage tightly with DNA, stabilizing it at the bottom of the β-ring helix. The net effect of a subunit losing registry with DNA at the top of the helix and a subunit gaining registry with DNA at the bottom of the helix is a compaction of the remaining ATP-bound subunits, generating force to translocate DNA 2 base-pairs towards the α-ring.

### Overall conserved features of translocation

The fundamental elements of the proposed mechanism are:

1. Nucleotide binding drives two competing effects: strong interaction with DNA that forces subunits into a helical configuration, while simultaneously promoting compaction of a subunit through internal conformational changes. Helicity promoted by interaction with DNA wins the competition.
2. ATP hydrolysis causes a subunit to disengage with DNA, which allows neighboring subunits to undergo ATP-induced compaction, translocating DNA past the hydrolyzing subunit.

Despite different aesthetic appearances of the models, these key features are strikingly similar to the fundamental features in the helical-to-planar model proposed for viral DNA packaging ATPases (13,25,30). Additionally, many AAA+ have been proposed to utilize a ‘hand-over-hand’ model of substrate translocation (31,32). In this model, ATP-bound subunits form a helical pitch as they track the biopolymer substrate. Hydrolysis at one end of the helix disengages a subunit from the substrate, and that substrate begins to reset to the other side of the helix. Translocation is accomplished by the remaining ATP-bound subunits shifting their positions as they maintain grip with the biopolymer substrate. Thus, it may be possible that these models are converging to the same fundamental aspects outlined above, and that differences emerge as system-specific constraints modulate the overall mechanism.

### Differentiating features of FtsK translocation and viral DNA packaging

While there may be mechanisms common to all translocating ring ATPases, each system has a unique evolutionary history and system-specific optimizations that must be considered. For this discussion, we will contrast the FtsK model to the viral DNA packaging helical-to-planar model.

The pore of the hexameric FtsK_αβ_ assembly is larger than the diameter of dsDNA, such that some subunits do not engage with DNA (18). This allows for FtsK_αβ_ subunits to reset from one end of the helix to the other during translocation without fighting against the translocated DNA. Thus, the pore size permits a continuous translocation mechanism as subunits are free to both translocate and reset simultaneously. On the other hand, viral DNA packaging ATPase rings are pentameric assemblies when attached to procapsids and actively translocating DNA, likely maintaining symmetry with the unique fivefold vertex of the capsids at which they assemble (13,33,34). The pore of the pentameric ATPase assembly is roughly the same diameter as dsDNA. Thus, any subunit resetting from one end of the helix to the other would have to fight against the translocated DNA. Accordingly, viral packaging ATPases have adapted burst-dwell packaging dynamics (11,30,35); DNA is translocated one helical turn in during a quick burst that is followed by prolonged dwells wherein no DNA is translocated as the entire motor resets. Partitioning translocation and motor reset phases ensures that each subunit is moving in the same direction and subunits do not compete against each other. Based on these observations, we propose that pore-size and the observation of burst-dwell dynamics can be used as discriminating whether a motor assembly utilizes a FtsK-like or a helical-to-planar translocation mechanism.

A second difference is the topology of the linker motif. In AAA+ and viral packaging ATPases, the connector between the globular N-terminal (ATPase) domain and C-terminal domains is a α-helical-rich lid subdomain that mediates a large amount of inter-subunit interactions (14,25). In the helical-to-planar and hand-over-hand models, rotation of the lid subdomain was proposed to be the force-generating mechanism (25,26). On the other hand, the connector between the α-/β-domains of the FtsK assembly is a short coil and does not interact with neighboring subunits, and thus an analogous mechanism cannot be the force-generating step. This difference is perhaps related to the overall goals of viral packaging ATPases/AAA+ and FtsK-like motors. Viral packaging ATPases/AAA+ motors often must produce high forces to accomplish their tasks, such as packaging DNA to near crystalline density inside of a viral capsid (36), or disassembling microtubules (14). A subunit being able to leverage neighboring subunits *via* the lid subdomain may help these motors generate high forces. On the other hand, FtsK-like motors do not translocate DNA against high forces but do translocate DNA an order of magnitude faster than viral packaging ATPases. Hence, FtsK-like motors may have adapted to favor mechanisms that promote fast translocation rather than those that generate high forces. Thus, the topology of the linker may be means to discriminate between mechanisms.

Lastly, hydrolysis is controlled by distinct means. In FtsK-like assemblies, hydrolysis is catalyzed *in trans* by two subunits that are helically offset from each other (18); viral DNA packaging ATPases are proposed to catalyze hydrolysis upon a planar alignment of two subunits (13,25). The difference in interfacial geometry at the time of ATP hydrolysis requires different sites for the canonical arginine finger within the subunits. Thus, the sequence location of the arginine finger may also be a means to discriminate between translocation models.

### Comparison to other simulation studies, caveats and future work

Because ATPase mechanochemistry is an inherently dynamic process, many researchers have attacked the problem with molecular dynamics simulations. Many of these results suggest similar dynamics and couplings as we propose in this current study. For instance, Kleinekathöfer et al. (37) found that two angles analogous to our ‘twist’ and ‘extension’ angles could describe the internal degrees of freedom of F_1_-ATPase subunits. Work on the SecA secretion complex also has strong parallels with this current work. Chen et al. (38) calculated the two-dimensional potential of mean force (PMF) of SecA as a function of an angle and distance between domains; this is analogous to our computed PMFs as a function of twist and extension. Further, Milenkovic and Bondar (39) demonstrated that a complex hydrogen-bonding network allows for communication between the signal peptide binding site and the nucleotide binding domain; this is similar to the connection between ATP binding and DNA gripping we propose herein. Lastly, Allen et al. (40) found that ATP binding and hydrolysis bias the direction of substrate translocation by affecting how the SecA/SecY assembly grips the polypeptide substrate, similar to how we propose that ATP hydrolysis promotes DNA translocation by controlling a subunit's grip on DNA in the FtsK system.

Like all the above molecular simulations, our study has some caveats and assumptions. In the *in vivo* system, the FtsK_αβ_ assembly has additional domains that attach to a membrane or provide additional DNA gripping residues. Thus, the dynamics of the FtsK_αβ_ assembly in isolation of these other domains may not necessarily represent those of the true *in vivo* complex; this is also a limitation to experimental studies where FtsK_αβ_ has been isolated from the larger assembly. Secondly, our predictions are fundamentally limited by the nature of fixed-charge molecular mechanics force fields. We tried to mitigate this by using an up-to-date AMBER force field and an accurate four-point water model. Still, fixed-charge descriptions are a balancing act, and their imperfections can be seen in systems where charge-charge interactions are critical. To our knowledge, polarizable descriptions have yet to be tested in large protein-DNA assemblies due to computational limitations. Lastly, while our simulation sampling is considerable by current standards (in the several microsecond range), it is still short compared to the timescales of many biologically relevant events. Thus, our predictions are likely missing dynamics and interactions, and hence mechanisms, that could be gleaned from longer timescale simulations. This could be remedied in the future as computational resources become more powerful and accessing the tens- or hundreds-of-microseconds timescale is readily available to researchers.

Despite these caveats, the validity of our study can be experimentally tested because we make specific residue-level predictions. We propose that Arg548 helps to selectively promote nucleotide exchange (Figures. 2 and 5). This prediction can be tested by mutating Arg548 and measuring the change in apparent *k*_off_. Another prediction is that γ-phosphate binding and ATP hydrolysis causes a subunit to grip or lose grip of DNA, whereas force generation is related to binding of the ATP/ADP nucleotide (Figures 1 and 4). This prediction can be tested by mutating key residues expected to interact primarily with the γ-phosphate (such as the phosphosensor Thr630/Gln631) and measuring the motor's affinity for DNA in the ATP- and ADP-bound states, similar to the experimental validation of our computational predictions in viral packaging ATPases (29). Alternatively, one could mutate residues primarily involved in adenosine binding (such as Phe695 or His675) and measure force generation with single-molecule studies. We would anticipate these variants would produce less force than the WT counterpart as is observed with viral packaging ATPases (41). We predicted that the helical pitch of subunits within the motor arises from their interaction with DNA (Figures. 3 and 4), in agreement with structural studies (18). This prediction can be tested by challenging the motor with a DNA/RNA hybrid (whose helical parameters are different to that of double-stranded DNA) and measuring how the motor adapts to accommodate this substrate. Such an approach has been recently applied to viral packaging ATPases (61) and demonstrated that their mechanochemical cycle is indeed governed by the helical pitch of the translocated substrate.

## DATA AVAILABILITY

All AMBER format topologies, initial coordinates, input run files, PDB format representative structures from k-means clustering, and .nmd files for visualizing the PCs of motion are included in the supplemental data.

## Supplementary Material

gkac668_Supplemental_Files
