# Supplementary material for: Molecular dynamics of DNA translocation by FtsK
Source: Nucleic Acids Res. 2022 Aug 10;50(15):8459–70. doi: 10.1093/nar/gkac668 (PMC9410874; doi:10.1093/nar/gkac668)
Supplement: gkac668_Supplemental_Files [file gkac668_supplemental_files.zip › ftsk_SI_revised_pdf.pdf]

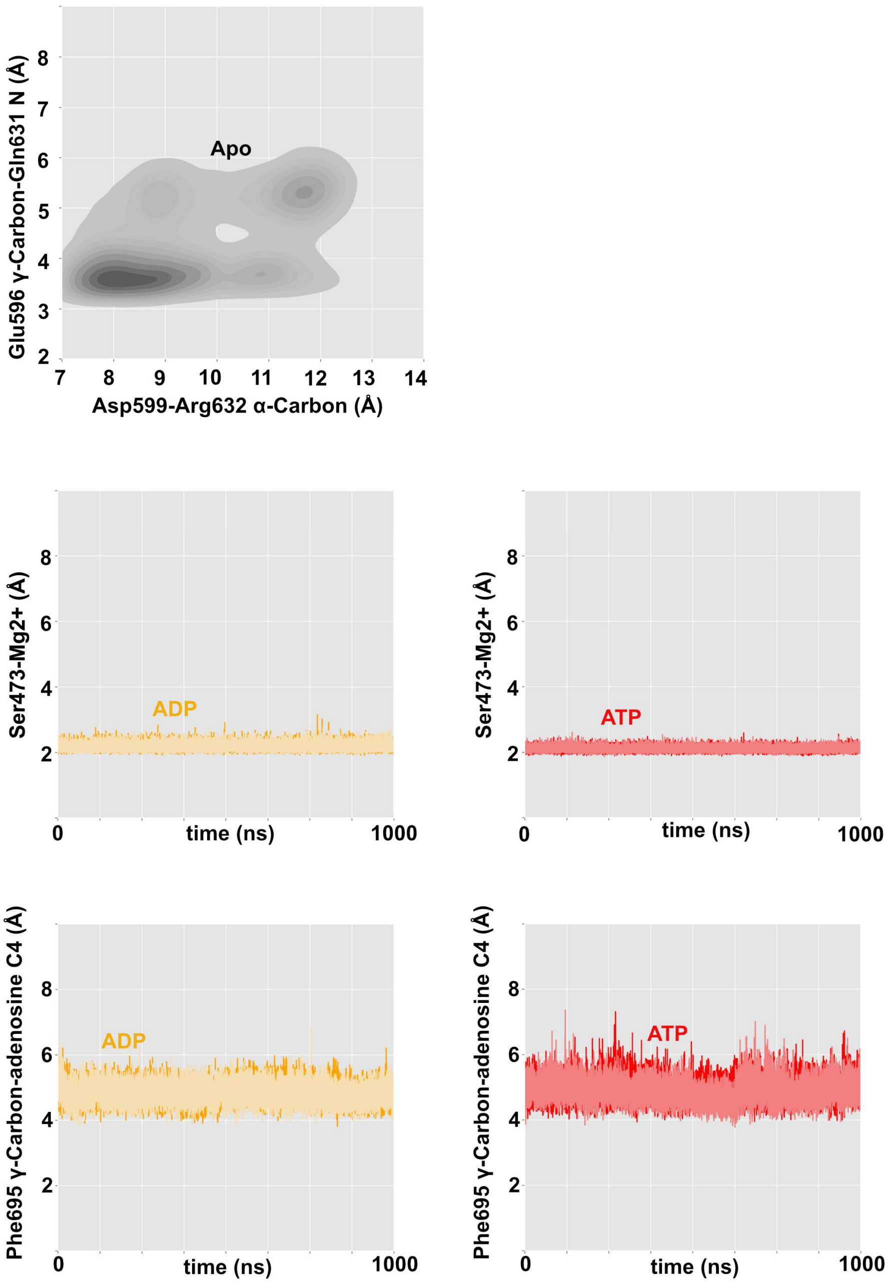

Figure S1. (Top) Distance distribution of the apo simulations shows that while the subunit is flexible, the majority of states sampled are similar to the ADP-bound state (main text **Fig. 1**). (**Below**) key distances in the ATP- and ADP-bound simulations are plotted to show the stability of the binding pose over the course of the simulation. The distance between the Walker A serine and the Mg^{2+} is shown, and the distance between the gamma-carbon of the Phe695 to a carbon in the adenosine ring of the bound nucleotide is depicted to show the stability of the π-stacking interactions.

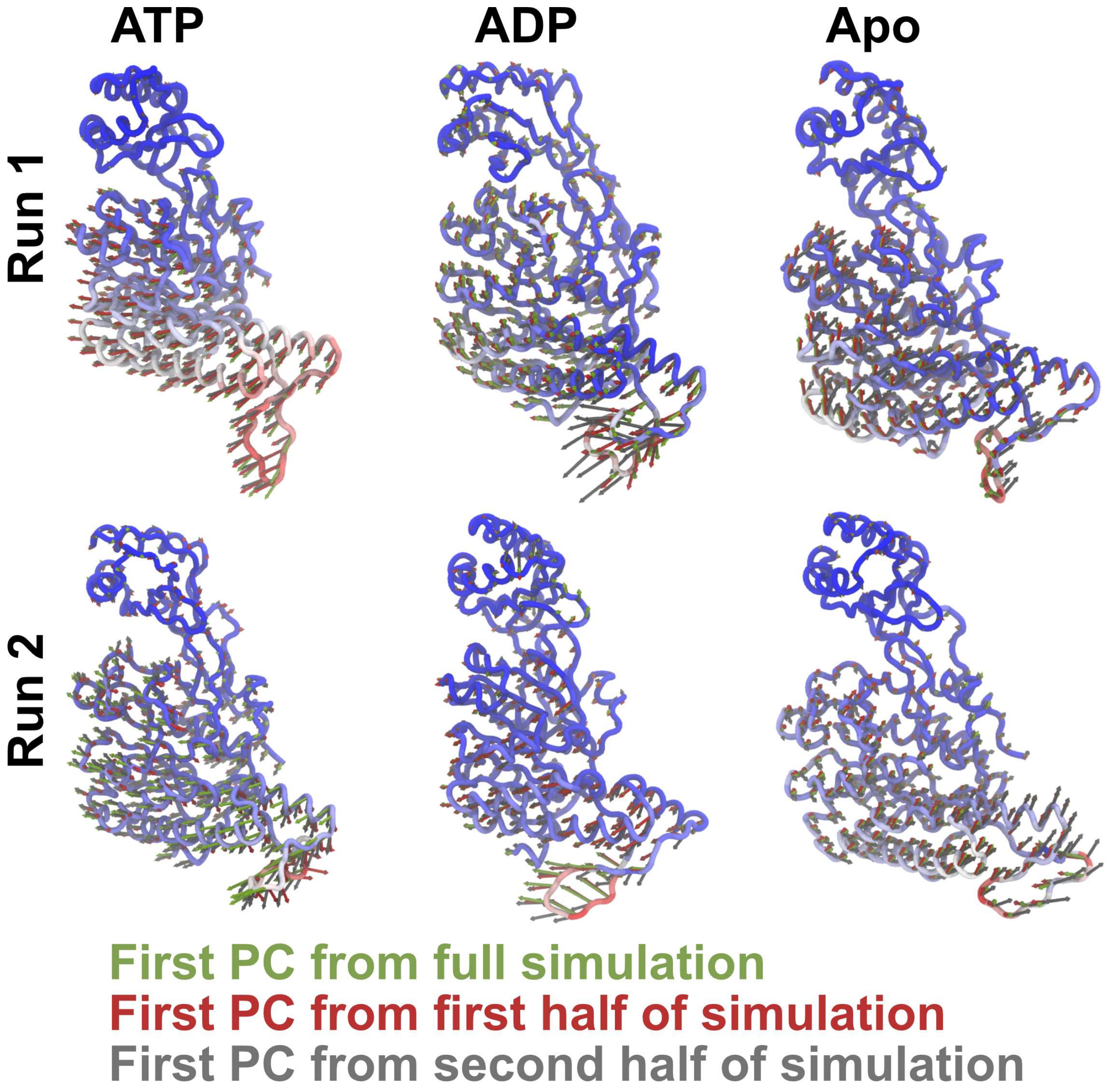

Figure S2. Convergence of principal components of motion. For all three nucleotide-bound states, PCs were calculated using the entire simulation (yellow-green arrows), the first half of the simulation (red arrows), and the second half of the simulation (gray arrows). In most cases there is excellent agreement between these projections, suggesting that our simulations have converged on the timescale considered.

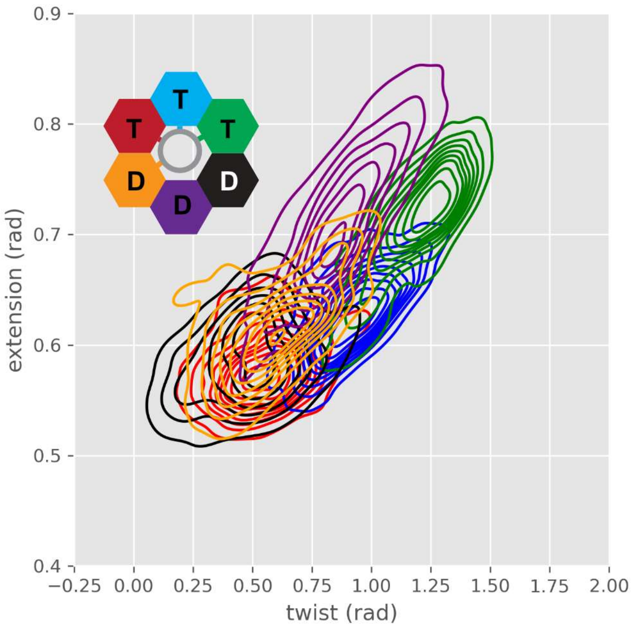

Figure S3. Twist-extension projection of the second hexamer simulation. In the second hexamer simulation, the DNA-gripping subunits (green, blue, and red) still form a helical pitch as they track the DNA substrate. Non-DNA gripping subunits exhibit higher flexibility.

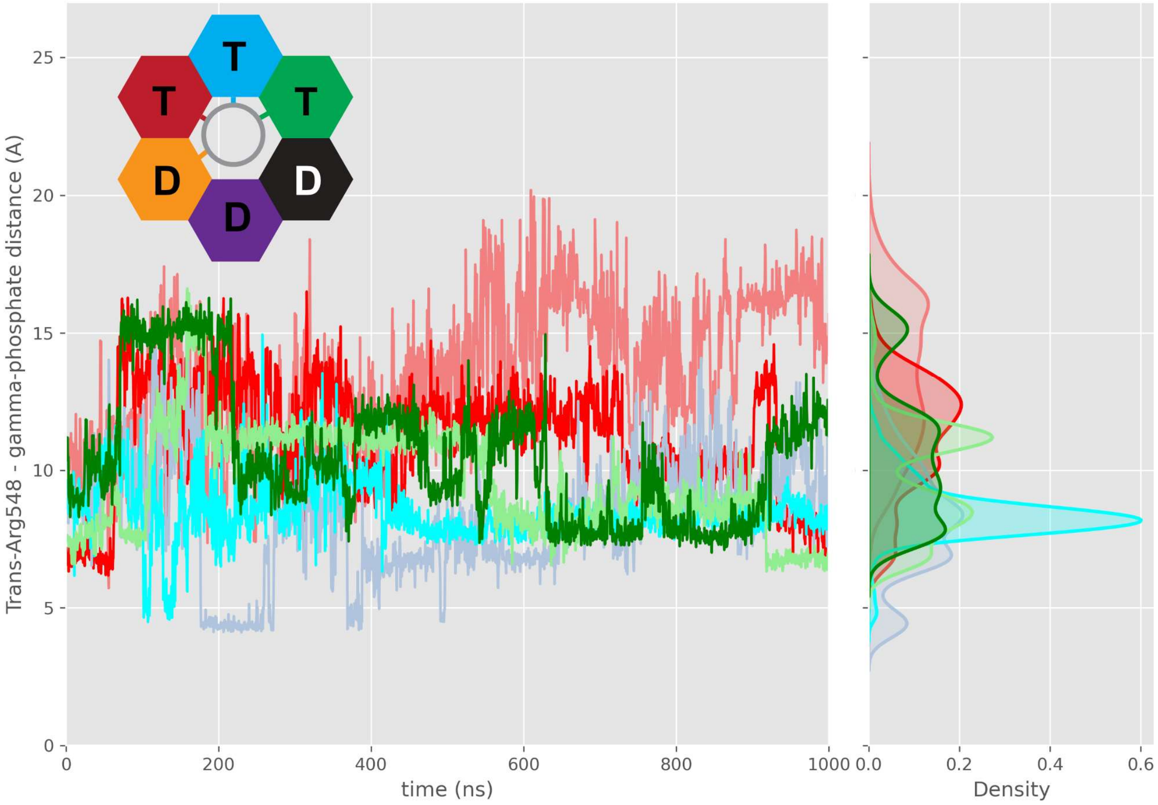

Figure S4. Arg548 distance to the γ-phosphate of ATP. Distances between the guanidine carbon of Arg548 and the γ-phosphate of ATP bound to the neighboring subunit. Colors correspond to the *cis*-acting binding site; pastel and dark designate duplicate simulations. Briefly and transiently, Arg548 is able to directly interact with the γ-phosphate of ATP in a neighboring subunit, demonstrating the ability to act as a catalytic moiety.

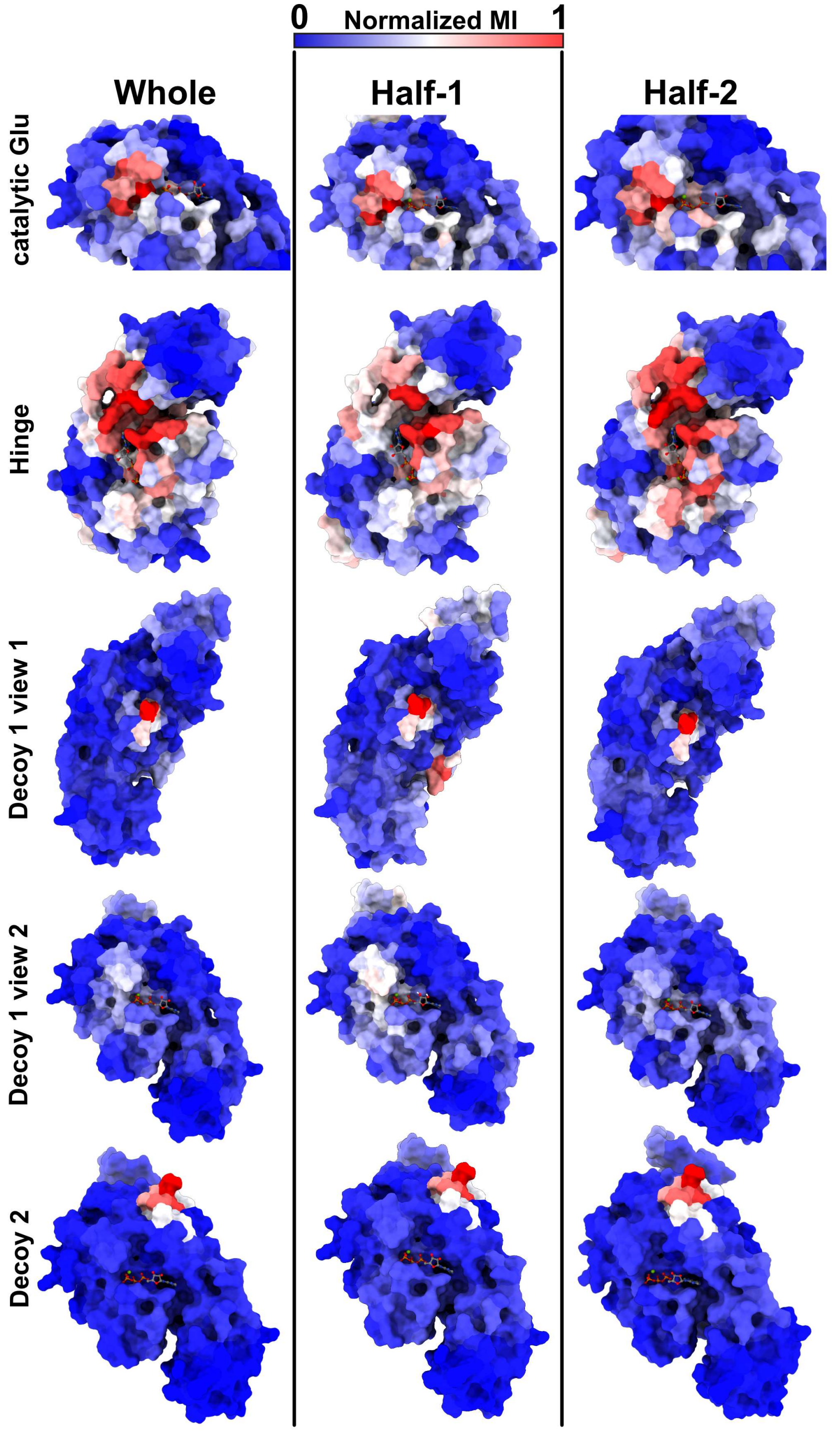

Figure S5. Mutual information by CARDS analysis. We calculated MI by CARDS using the whole dataset and both half data sets (indicated by the column headers). In general, these half data sets show good agreement with the whole data set, suggesting convergence on the timescales considered. Target site analysis was performed relative to the motifs labeled in the rows. To test for spurious MI calculations, we included two decoy target sites. Decoy 1 is on the opposite side of the FtsK monomer as the ATPase active site close to *trans*-acting residues, and shows little-to-no normalized MI with the active site. Decoy 2 is close to the active site and again shows little-to-no normalized MI with the active site.

Movie S1. First principal component of motion of the first ATP-bound monomer simulation.

Movie S2. First principal component of motion of the second ATP-bound monomer simulation.

Movie S3. First principal component of motion of the first ADP-bound monomer simulation.

Movie S4. First principal component of motion of the second ADP-bound monomer simulation.

Movie S5. First principal component of motion of the first apo monomer simulation.

Movie S6. First principal component of motion of the second apo monomer simulation.

Movie S7. First principal component of motion of the hexamer simulations. After aligning about the α-ring, PCA was performed on the entire FtsK assembly. Subunits in the β-domain helix are depicted. As the ATP-bound subunits (red, cyan, and green) rock their β-domain upwards, in the direction of DNA translocation, the ADP bound subunit (orange) rocks oppositely, beginning to lose grip of DNA and reset towards the bottom of the β-domain helix.

Movie S8. First principal component of motion of the hexamer simulations. A top-down view is shown. Subunits that do not grip DNA (purple and gray) are more flexible than the rest of the assembly. Their rotations directly modulate the ATPase active sites in the orange subunit (predicted to be the catalytic interface), and the gray subunit (predicted to be the site of nucleotide exchange).

Movie S9. First principal component of motion of the hexamer simulations. After aligning about the α-domain of a single subunit, PCA was performed. The primary mode of motion is a rocking and shearing, corresponding to the extension and twist angles defined as our collective variables.
